# Artificial Intelligence in Breast Reconstruction: A Narrative Review

**DOI:** 10.3390/medicina61030440

**Published:** 2025-02-28

**Authors:** Andrei Iulian Rugină, Andreea Ungureanu, Carmen Giuglea, Silviu Adrian Marinescu

**Affiliations:** 1 Department of Plastic and Reconstructive Surgery, “Bagdasar-Arseni” Emergency Hospital, University of Medicine and Pharmacy “Carol Davila”, Blvd. Eroii Sanitari Nr. 8, Sector 5, 050474 Bucharest, Romania; andreir180@gmail.com (A.I.R.); silviu.marinescu@umfcd.ro (S.A.M.); 2 Department of Plastic and Reconstructive Surgery, University of Medicine and Pharmacy “Carol Davila”, Blvd. Eroii Sanitari Nr. 8, Sector 5, 050474 Bucharest, Romania; giugleacarmen@yahoo.com

**Keywords:** artificial intelligence, robotic surgery, machine learning, virtual reality, augmented reality, microsurgery/artificial intelligence, deep learning, breast reconstruction

## Abstract

Breast reconstruction following mastectomy or sectorectomy significantly impacts the quality of life and psychological well-being of breast cancer patients. Since its inception in the 1950s, artificial intelligence (AI) has gradually entered the medical field, promising to transform surgical planning, intraoperative guidance, postoperative care, and medical research. This article examines AI applications in breast reconstruction, supported by recent studies. AI shows promise in enhancing imaging for tumor detection and surgical planning, improving microsurgical precision, predicting complications such as flap failure, and optimizing postoperative monitoring. However, challenges remain, including data quality, safety, algorithm transparency, and clinical integration. Despite these shortcomings, AI has the potential to revolutionize breast reconstruction by improving preoperative planning, surgical precision, operative efficiency, and patient outcomes. This review provides a foundation for further research as AI continues to evolve and clinical trials expand its applications, offering greater benefits to patients and healthcare providers.

## 1. Introduction

Artificial intelligence (AI) is an emerging field that harnesses computer technology to investigate and advance theories, methodologies, techniques, and application systems aimed at simulating, extending, and enhancing human intelligence [1]. AI is transforming a vast majority of industries, ranging from finance and manufacturing to transportation and customer service. AI chatbots, self-driving cars, industrial automation, and recommendation systems on streaming platforms are some examples of how machine learning and deep learning models can analyze large sets of data, recognize potential patterns, and optimize decision-making in real time. In healthcare, AI is rapidly developing, becoming a valuable tool and improving diagnostics, treatment planning, and patient outcomes across multiple medical or surgical fields [2,3]. Due to its versatility, AI has found numerous applications in medicine, particularly in areas such as image processing, computer vision, artificial neural networks, machine learning, convolutional neural networks, and deep learning [3].

Healthcare systems worldwide face substantial challenges in delivering efficient and high-quality care to diverse populations. Aging populations, the growing burden of chronic diseases, and permanently rising healthcare costs are factors that should motivate governments and insurance providers to optimize the healthcare sector [4]. AI is revolutionizing healthcare by optimizing workflows and enabling medical professionals to provide superior care, resulting in better patient outcomes. For individuals, AI has the potential to expand access to healthcare services, providing greater satisfaction and compliance with treatments [5]. In recent years, integration of AI in the healthcare sector has seen impressive advancements. Machine learning algorithms are now capable of detecting disease in early stages, with AI-assisted tools capable of achieving diagnostic accuracy comparable to clinicians [6]. In surgery, robotic platforms like the Da Vinci surgical system enhances precision and outcomes and reduce postoperative morbidity [7]. AI also reduces the bureaucratic burden on health care providers through natural language processing, which improves medical documentation and administrative efficiency [8]. While it has the potential to improve the healthcare system, the integration of AI into healthcare encounters several challenges: data security and privacy, a lack of standard ethical guidelines, algorithm bias, and educational gaps. To ensure an effective and ethical integration of AI in the healthcare system, these downfalls must be meticulously examined and addressed [9].

Surgeons in the field of plastic surgery, using various techniques (alloplastic materials or autologous tissues) restore breast appearance postmastectomy [10]. In general, the rate of success after surgery and the overall outcome is strongly dependent on the surgeon’s training and experience in planning and performing the reconstruction. Although AI has been explored in plastic surgery and other fields [11,12], its data processing capabilities and complementary technologies have the potential to enhance various aspects of the surgical craft, making it equally applicable to breast reconstruction [13]. This review examines the current literature to offer a thorough overview of the applications of AI and complementary technologies in reconstructive breast surgery.

## 2. Artificial Intelligence Applications in Breast Reconstruction

### 2.1. Preoperative Planning

Extensive and thorough preoperative planning is key for any successful breast reconstruction. AI-assisted imaging tools offer in-depth 3D reconstructions of patient anatomy that can be helpful in any type of reconstruction—primary, when the breast is reconstructed in the same surgery as the mastectomy and lymphadenectomy, or secondary, which occurs at a later time. Studies indicate that AI algorithms significantly improve the accuracy of Magnetic Resonance Imaging (MRI) and Computer Tomography (CT) scan interpretations, assisting in the detection and classification of tumors or identifying and localizing key anatomical structures for surgical planning. AI systems can be trained to identify and classify relevant anatomical landmarks, potential lesions, vessels, and other important features on machine-generated scans [14].

#### 2.1.1. Preoperative Imaging

In planning a breast reconstruction using the deep inferior epigastric perforator (DIEP) flap, CT-angiography (CTA) is employed to identify and select the appropriate perforator [15]. However, this process can be challenging, requiring specialized knowledge, which may be difficult for young physicians. AI can enhance to some degree the diagnostic accuracy and reporting speed. Indeed, Civik J. et al. highlighted the role of AI in predicting vascular anatomy in the abdominal region and highlighted the importance of optimal perforator selection, aiding better planning for DIEP flap procedures, shortening time-consuming human analysis, and potentially expediting surgical procedure time [14,16,17]. Likewise, Mavioso C. et al. compared the results of manual and semi-automatic identification with the intraoperative dissection of the perforator vessels in 40 CT-angiographies from 40 patients that were proposed for immediate or delayed breast reconstruction. The algorithm used matched manual methods in accuracy for larger vessels and also reduced preoperative analysis time by two hours per patient, with clinically insignificant vessel location discrepancies [13].

Beyond preoperative planning, deep learning algorithms, such as those using U-net architecture, can help reduce clinician labor and variability in image analyses by advancing automatic segmentation. AI technologies can enhance detection capabilities and support clinical decision-making, offering efficient tumor size and volume measurement [18]. The data from 48 cohort studies on breast imaging revealed impressive detection accuracy across various modalities. Mammography demonstrated high diagnostic performance, with a measure of accuracy (AUC) close to 0.87, proving its ability to distinguish between healthy and abnormal tissues. Ultrasound showed better results, with an AUC around 0.91, reflecting even greater precision. Similarly, MRI and digital breast tomosynthesis (DBT) achieved comparable accuracy levels, 0.87 and 0.91, respectively. These findings highlight the effectiveness of these imaging methods in detecting breast abnormalities, providing confidence in their diagnostic capabilities [6]. While nuclear medicine imaging modalities like PET (Positron Emission Tomography) or scintigraphy are less efficient for early-stage diagnostic evaluation of breast cancer compared to mammography, MRI, DBT, or ultrasound, they play an important role in the detection and classification of axillary lymph nodes and distant staging. A review by Balkenende et al. [19] highlights the use of convolutional neural networks by various authors to improve and determine detection rates using nuclear imaging (Table 1).

#### 2.1.2. Risk Stratification and Decision Support

Breast reconstruction, utilizing either autologous tissues or alloplastic materials, can be a significant source of morbidity for patients. This morbidity stems from complications that may arise either immediately postoperatively or at a later stage. A wide range of complications can manifest following breast reconstruction, with some being more prevalent depending on the selected reconstructive technique. The most common complications include flap venous congestion, capsular contracture, seroma, local wound infection, and skin necrosis [20,21]. In response to these challenges, O’Neill et al. have developed a machine learning model to predict the risk of flap failure in patients undergoing DIEP breast reconstruction. By analyzing patient characteristics and surgical technique factors, the model identified risk factors such as obesity, smoking, and timing of reconstruction, helping predict whether postoperative flap failure might occur [22]. Similarly, Yunchan Chen et al. used a neural network model to predict capsular contracture after two-stage expander/implant-based reconstruction. The model analyzed specific risk factors and provided surgeons with a percentage-based risk assessment of developing capsular contracture, potentially guiding the surgeon to opt for a flap-based reconstruction [23]. Furthermore, Yi-Fu Chen used a machine learning model to predict the need for postmastectomy radiation therapy after immediate breast reconstruction. By analyzing preoperative patient characteristics, the model provided personalized predictions about the need for post-reconstruction radiation therapy, which can negatively impact alloplastic reconstructions, therefore assisting surgeons in choosing the time and type of reconstruction [24].

#### 2.1.3. Outcome Prediction

Artificial intelligence offers the capability to predict cosmetic outcomes during the preoperative planning of breast surgery, giving both clinicians and patients a clearer understanding of potential results. For instance, machine learning proved useful in creating formulas for predicting breast volume using anthropometric measurements [25]. Additionally, deep learning models have been used to assess breast volume and density on MRI scans, which can guide the selection of appropriate implants for reconstruction [18]. Building on this, Chartier et al. explored a neural network trained on real clinical imagery, which proved highly effective in generating preoperative simulations that closely aligned with actual postoperative appearances. This approach can be particularly valuable in contralateral breast surgery, as it helps achieve optimal symmetry with the newly reconstructed breast [26]. Also, Didzbalis et al. studied patient concerns about mastopexy using data from a social media site and machine learning techniques to help surgeons address common concerns during consultations and improve overall patient satisfaction [27]. In addition to these applications, machine learning models can improve the informed consent process by giving patients more accurate, personalized predictions about recovery and risks. This helps manage expectations and provides better preoperative guidance, which is directly linked to higher satisfaction after surgery [28,29].

### 2.2. Intraoperative Guidance

When speaking of the operating room, AI systems can overlay crucial information, like the location of blood vessels and tissues, directly onto the surgeon’s view. AI can extend its usefulness in the operating setting, using advanced computer vision systems, augmented reality technologies and surgical robots (Da Vinci), in some cases offering important guidance and enhancing surgical precision [30]. For now, AI has not reportedly been used in the operating room for breast reconstruction, but some authors reported the usage of AI to define and project safe dissection planes in real time while performing gastrectomies [31]. For breast reconstruction, this could involve recognizing connective tissue layers and avascular zones to guide precise and safe surgical movements during lymph node dissection or tissue dissection during flap harvest. Although not related to breast surgery, Russell et al. demonstrated enhanced surgical precision, reducing morbidity and improving postoperative outcome using a five-axis robot (IBM 7576) equipped with an added pitch axis, force sensor, and surgical cutting tools [32].

Advanced robotic systems, such as the Da Vinci robot, have been utilized to anchor acellular dermal matrices, flap harvest, and perform microsurgical anastomoses with unprecedented accuracy [33,34,35]. Complementary to AI, augmented reality has emerged as a valuable tool for intraoperative visualization, enabling surgeons to overlay 3D anatomical data onto the surgical field for precise navigation. These advancements not only optimize technical performance but also ensure better postoperative results through enhanced surgical precision and reduced fatigue-related errors [36]. Technologies like Microsoft’s HoloLens allow surgeons to overlay CTA images directly onto the surgical field, enhancing their understanding of the anatomy before making incisions or performing tissue dissections [37]. Looking ahead, the field of intraoperative use of AI in robotic surgery has seen rapid growth in the last years but mainly remains in preclinical stages, with no clinical studies demonstrating the application of the technology to all the tasks humans perform. Current technologies focus on isolated tasks such as robot control and instrument tracking, operating at low autonomy levels [7].

### 2.3. Postoperative Care and Monitoring

Postoperative complications are a major concern in breast reconstruction. AI is transforming postoperative care by enabling continuous monitoring and early detection of possible complications. Myung et al. validated the use of machine learning models to predict donor-related complications in 568 patients undergoing muscle-sparing TRAM flap breast reconstruction. They demonstrated that AI technologies can effectively assess the risk of donor site related complications in reconstructive breast surgeries. Among the machine learning methods evaluated, they found that neuralnet achieved the highest accuracy (81%) [38]. AI can also be used to predict postoperative complications such as periprosthetic infection and the need for explantation with greater accuracy compared to traditional methods. By analyzing specific factors and surgical variables, these models provide clinicians with actionable insights to tailor interventions and optimize recovery [39].

Artificial intelligence, especially large language models (LLMs) like ChatGPT-3.5, -4 and Gemini, were evaluated in providing postoperative care advice in plastic surgery. LLMs have shown promise in offering personalized advice to patients on wound care, activity restrictions, symptom monitoring, and the early recognition of complications [40].

The effective postoperative monitoring of flaps is crucial for preventing flap failure and ensuring successful reconstruction, with physician involvement with direct clinical observation being the gold standard [41]. Kim et al. developed an AI-based automated system for free flap monitoring capable of appreciating flap perfusion based on photographs. The system demonstrated potential for efficient monitoring with minimal human involvement, reducing the burden on medical staff [42].

Symmetry analysis after breast reconstruction is time-consuming for clinicians, and AI has the ability to quickly process and analyze symmetry, helping to monitor changes in time and allowing clinicians to assess how well the reconstruction matches preoperative goals [43].

### 2.4. Personalized Treatment Plans

AI’s ability to analyze large datasets allows for personalized treatment plans, tailoring reconstruction strategies to individual patient needs [44,45]. In the future, AI could play a strong role in recommending the optimal reconstructive approach depending on the patient’s characteristics and preferences. Also, it could refine choices such as the donor site for flaps, the recipient vessel, expander or implant selection, and surgical techniques [46].

Giving AI’s ability to enhance various aspects of breast reconstruction, potentially improving aspects like preoperative planning, preoperative imaging, risk stratification and decision support, outcome prediction, intraoperative guidance and postoperative care and monitoring, a theoretical comparison of traditional versus AI-assisted breast reconstruction in terms of accuracy, time efficiency, overall outcomes and costs is presented in Table 2. 

### 2.5. Enhancing Educational Training and Scientific Research

AR technologies have surfaced in surgical training bringing important benefits to medical trainees [47]. Although primarily explored in urology for penile implant placement and orthopedics for acetabular cup orientation, the approach demonstrates the potential of integration in breast reconstruction, providing enhanced visualization and improved training for residents [48,49]. Academic institutions like Stanford Medicine have already integrated AR technologies into practical surgical training [50]. In terms of research, when used with proper caution and adherence to specific guidelines, LLMs can have a significant impact the field, providing volume to ideas, guiding new researchers, analyzing extensive patient data to identify patterns, and potentially creating new insights or hypotheses for future researchers [51,52]. Machine learning can also be employed to analyze large datasets, identifying relationships between patient subgroups, ultimately improving clinical decision-making [53].

## 3. Discussions

AI integration into breast reconstruction is a rapidly expanding domain, and the present review aims to provide a strong foundation about the current state of the matter for surgeons and researchers, examining potential benefits, challenges and future directions. Like with any expanding scientific field, accumulating and refining evidence will take time as the overall knowledge on the subject continues to grow. Although promising, challenges such as inaccuracies, algorithm transparency, vulnerability to hacking or breaches, and integration into clinical practice remain significant drawbacks to permanent integration. Also, the use of AI in healthcare can pose significant risk, and adherence to specific recommendations is mandatory [54]. One of the most critical areas where AI could pose significant risk is inside the operating theater. A major concern is the potential for system errors, which tragically could lead to incorrect surgical decisions or even delays in response. For example, AI-assisted robotic systems use real-time data processing and machine learning algorithms, which, if trained on unbalanced or unrepresentative datasets, could result in poor identification of key anatomical landmarks and structures resulting in inaccurate robotic gestures during surgery. Additionally, latency in remote or telerobotic surgery has the potential to generate timing errors, compromising patient safety. The prevention of these risks involves the robust validation of AI systems through extensive clinical trials and permanent human oversight [55,56]. Concerns about AI in surgery extend beyond intraoperative risks to potential job displacement for surgeons and even across the entire healthcare industry. Being a rather rapidly expanding field, complete automation could be possible in the near future, reducing the demand for human intervention and potentially impacting the job market, limiting training opportunities [57]. AI misuse in the health care sector, especially in surgical domains like breast reconstruction, could lead to serious complications and ultimately to significant legal concerns. Determining responsibility in such cases can be challenging and should be one of the main topics of debate for governments and regulatory agencies [56]. Future studies should focus on creating transparent, understandable AI systems and address ethical concerns, such as patient data privacy and consent.

In recent years, AI has been widely adopted across fields like computer science, finance, data security, social media, travel and transportation, the automotive industry, and education [58], highlighting the need for its integration into healthcare in the near future. This means that AI has the potential to improve various areas of breast reconstruction (Table 3) and the entire health care system, with breast reconstruction being a core subject worldwide [59].

Advances in other surgical specialties hold significant potential for integration into breast reconstruction, a field that remains relatively underexplored in current medical research. For now, robotic-assisted microsurgery has been mentioned to enhance precision and dexterity in complex procedures like DIEP flap reconstructions, and the integration of AI and complementary technologies may expand the potential for surgical planning and execution, providing optimized outcomes [33,47,60]. Additionally, the integration of computer vision could offer further benefits, paving the way for autonomous robots in the future [61]. Further applications in the field include AR technologies, which potentially offer benefits for surgical planning, execution and overall outcomes. HoloLens has demonstrated its ability to overlay CTA image information onto patients, helping in the identification and planning of perforator dissection [37,62]. Likewise, MRI and CT angiography images have also been utilized with AR headsets to assess breast morphology and abdominal perforator visualization, offering valuable insights for planning reconstructive surgery with microsurgical transfers [63,64]. However, as the technologies seem to become more precise in improving surgical precision, integration into clinical practices faces challenges that stem from the need for extensive training and costs associated with advanced equipment [65]. Other potential applications of AI include integration in 3D printing technology, which could potentially enable detailed simulations of breast models, optimizing surgical planning of flaps and aesthetic outcomes [66]. Also, as stated in the preoperative planning section, deep learning models have been used to assess breast volume and density on MRI scans, providing surgeons valuable insights for implant selection. Given this ability to evaluate different tissue types across different imaging studies, AI can potentially be used in the future for optimizing fat grafting strategies. By analyzing volume retention and tissue viability across different imaging techniques for assessing fat grafts, it can play a crucial role in developing personalized treatment plans for hybrid breast reconstructions [18,67].

For the present time, it is clear that AI cannot fully replace the surgical team and is not capable of complete autonomy like humans, but it is clear that the integration into various aspects of the surgical practice has the potential to contribute to its overall improvement. The future of breast reconstruction will involve closer collaboration between surgeons, researchers, and computer technologists, creating innovations that are both clinically relevant and technically advanced. Similarly, the implementation of AI technologies requires dedicated training for both surgeons and auxiliary medical staff, as well as the conduction of clinical trials to ensure patient safety [68].

The present review manuscript provides a comprehensive analysis of the current available literature on AI applications in breast reconstruction, providing a valuable resource for surgeons and researchers in this field. Mainly, the review tries to connect knowledge from various specialties, including surgery, radiology, computer science, complementary technologies, and robotics, and highlights the benefits AI can provide in surgical planning and overall patient outcomes. Mentioning the latest advancements in AI and complementary technologies, the manuscript offers insights into how these innovations can benefit the practice of breast reconstruction. Clearly, several limitations must be acknowledged. Firstly, as a narrative review, this article lacks quantitative analysis included in systematic reviews or meta-analyses. Secondly, while the review includes many studies, certain aspects or applications were not fully addressed to not derail from the scope of this review. Thirdly, the exponential growth of technological advancements in AI means that some of the cited manuscripts and technologies could become outdated quickly. Additionally, there is an immense lack of clinical trials, greatly reducing the amount of high-quality data that could have been mentioned in multiple areas of the review.

Finally, AI and complementary technologies hold significant potential for advancements in breast reconstruction, enhancing both clinical practices and learning processes, potentially lowering healthcare expenses [69,70].

## 4. Conclusions

AI in the field of breast reconstruction could see rapid growth and has the potential to metamorphose current techniques by assisting in preoperative planning, increasing surgical precision, shortening operative times, providing predictive models for postoperative complications, and overall improving outcomes and patient satisfaction. The present review has the purpose of providing a framework for further research in the applications of artificial intelligence in breast reconstruction. As AI evolves and more prospective clinical trials are conducted, its applications in breast reconstruction will likely expand, providing even greater benefits to patients and healthcare providers.

## Figures and Tables

**Table 1:** Studies exploring the use of AI in nuclear imaging diagnosis [19].

Study	Technology Used	Application	Findings
Weber et al.	Whole-body PET scans and convolutional neural network (CNN)	Lesion detection and segmentation	39% sensitivity for lesion detection
Papandrianos et al.	Whole-body scintigraphy and CNN	Malignancy classification	92% accuracy
Li et al.	PET/CT and 3D CNN	Metastatic lymphadenopathy diagnosis	Improved clinician sensitivity by 7.8% without affecting specificity

**Table 2:** Theoretical advantages of AI-assisted breast reconstruction over Traditional Techniques.

Parameter	Traditional Breast Reconstruction	AI-Assisted Breast Reconstruction
Accuracy	Higher variability in outcomes due to human factors (surgeon skill)	AI tools can potentially assist in precise planning, improving aesthetic outcomes and symmetry
Time Efficiency	Longer operative times due to complex planning and execution	Potentially shorter procedure times, AI can optimize surgical planning and predict complications
Patient Outcomes	Variable satisfaction, with some patients experiencing dissatisfaction due to aesthetic results	Has the potential to provide higher patient satisfaction due to more predictable results
Costs	Higher costs due to longer operative times and potential need for multiple procedures	Initially, the cost could exceed traditional methods but potentially reduce overall costs that stem from complications and secondary surgeries.

**Table 3:** Manuscript’s key studies, methodology used, and findings.

Study	Methodology	Key Findings
O’Neill et al. (2020) [22]	Machine learning model for predicting flap failure	Identified obesity, smoking, and timing as major risk factors
Kim et al. (2024) [42]	AI-based free flap monitoring system	Efficient perfusion monitoring
Chartier et al. (2022) [26]	Neural network for preoperative breast simulations	Accurately predicted postoperative appearance
Myung et al. (2021) [38]	Machine learning for donor site related complications	81% accuracy in prediction
Mavioso et al. (2020) [13]	AI-assisted identification of perforators for microsurgical reconstruction	Reduced preoperative analysis by two hours per patient
Y-F Chen et al. (2024) [24]	Machine learning model for postmastectomy radiation therapy prediction	Provided personalized radiation therapy recommendations
Hassan et al. (2023) [39]	AI modeling for periprosthetic infection prediction	Improved prediction accuracy for implant complications
Chen et al. (2023) [23]	Neural network predicting capsular contracture	Provided percentage-based risk assessment
Kenig et al. (2024) [43]	AI-based breast symmetry evaluation	Automated symmetry analysis for postoperative assessment

## Data Availability

Data are available from the corresponding author upon reasonable request.
